# Case Report: Pseudomeningeosis and Demyelinating Metastasis-Like Lesions From Checkpoint Inhibitor Therapy in Malignant Melanoma

**DOI:** 10.3389/fonc.2021.637185

**Published:** 2021-04-15

**Authors:** Teresa Schmidt, Sied Kebir, Elisabeth Livingstone, Andreas Junker, Stefan Zülow, Lazaros Lazaridis, Christoph Oster, Eleftheria Chorti, Daniela Pierscianek, Refik Pul, Kathy Keyvani, Ulrich Sure, Martin Stuschke, Christoph Kleinschnitz, Björn Scheffler, Lisa Zimmer, Martin Glas

**Affiliations:** ^1^ Department of Neurology, Division of Clinical Neurooncology, University Hospital Essen, Essen, Germany; ^2^ West German Cancer Center (WTZ), University Hospital Essen, University Duisburg-Essen and German Cancer Consortium (DKTK), Partner Site University Hospital Essen, Essen, Germany; ^3^ German Cancer Research Center (DKFZ)-Division of Translational Neurooncology at the WTZ, DKTK Partner Site, University Hospital Essen, Essen, Germany; ^4^ Department of Dermatology, University Hospital Essen, Essen, Germany; ^5^ Institute of Neuropathology, University Hospital Essen, Essen, Germany; ^6^ Centre of Neurology-Radiology, University Hospital Bonn, Bonn, Germany; ^7^ Department of Neurosurgery, University Hospital Essen, Essen, Germany; ^8^ Department of Radiotherapy, University Hospital Essen, Essen, Germany

**Keywords:** melanoma, immune checkpoint inhibitors, immune-related adverse events, leptomeningeal metastasis, pseudomeningeosis

## Abstract

Immune checkpoint inhibitors (ICIs) have considerably expanded the effective treatment options for malignant melanoma. ICIs revert tumor-associated immunosuppression and potentiate T-cell mediated tumor clearance. Immune-related neurologic adverse events (irNAEs) manifest in the central (CNS) or peripheral nervous system (PNS) and most frequently present as encephalitis or myasthenia gravis respectively. We report on a 47-year old male patient with metastatic melanoma who developed signs of cerebellar disease five weeks after the start of ICI treatment (ipilimumab and nivolumab). Magnetic resonance imaging (MRI) of the brain and spine revealed multiple new contrast enhancements suggestive of parenchymal and leptomeningeal metastasis. Cerebral spinal fluid (CSF) evaluation showed a lymphomononuclear pleocytosis in the absence of tumor cells. Subsequent stereotactic brain biopsy confirmed demyelinating disease. High-dose corticosteroid treatment resulted in immediate improvement of the clinical symptoms. MRI scans and CSF re-evaluation were conducted six weeks later and showed a near-complete remission. The strong resemblance to neoplastic CNS dissemination and irNAEs is a particularly difficult diagnostic challenge. Treating physicians should be aware of irNAEs as those can be effectively treated with high-dose steroids.

## Introduction

Treatment with immune checkpoint inhibitors (ICIs) has revolutionized cancer treatment in the last decade (1). In melanoma, combining ICIs, such as ipilimumab – a monoclonal antibody directed against cytotoxic lymphocyte associated protein-4 (CTLA-4) – and nivolumab – a monoclonal antibody targeting programmed cell death 1 (PD-1) – has led to a significant improvement of treatment outcome (1, 2). ICIs are thought to reverse tumor-associated immunosuppression and to potentiate T-cell mediated tumor clearance (3). Treatment with ICIs can induce specific adverse events due to increased immunostimulation (3). NAEs occur early into the treatment with ICIs. Of those, irNAEs are rare and may present as unspecific symptoms including headache, vomiting or dizziness. More severe presentations comprise polyradiculitis, myasthenia gravis, encephalitis or demyelinating disease (4, 5). The incidence of irNAEs of any grade varies between 4% for ipilimumab and 6% for nivolumab and 12% for the combination of nivolumab and ipilimumab (5). Notably severe irNAEs only account for 0.8% of the reported irNAE with combinatorial treatment (5).

We here demonstrate the emergence of leptomeningeal, parenchymal and periventricular contrast-enhancing CNS lesions under combined nivolumab and ipilimumab treatment mimicking leptomeningeal and brain metastases in a patient with malignant melanoma.

## Case Presentation

**Figure 1:**
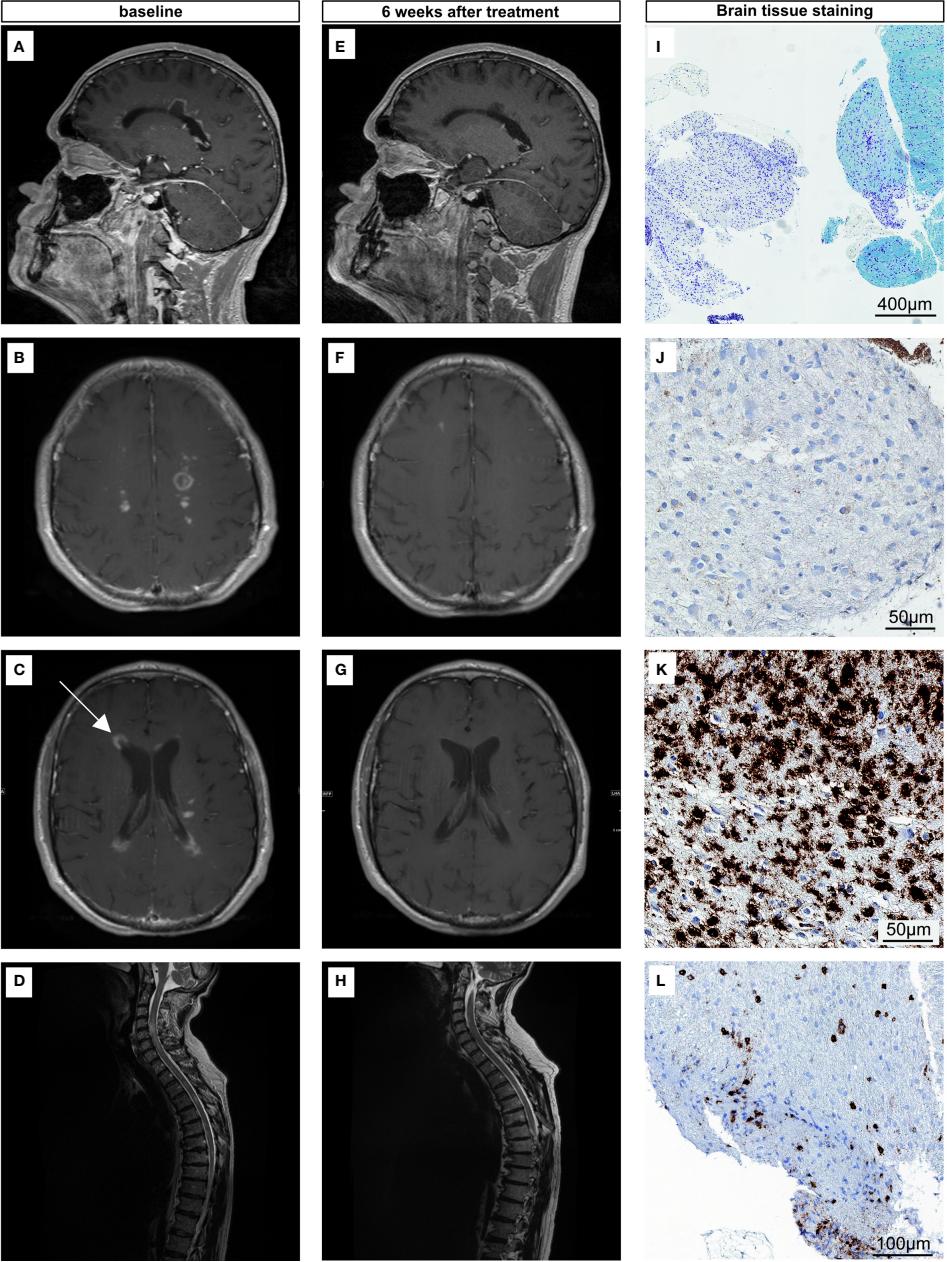
**(A–D)** MR-Images of the brain and spine showing multiple periventricular, parenchymal and leptomeningeal contrast enhancements at the time of diagnosis. T1-weighted contrast-enhancement MRI sequence of the brain **(A–C)** and T2- weighted MRI sequence of the spine **(D)**. Arrow pointing to the site of biopsy **(C)**. **(E–H)** MR-Images 6 weeks after high-dose steroid treatment showing near complete remission. T1-weighted contrast-enhancement MRI sequence of the brain after steroid treatment **(E–G)** and T2- weighted MRI sequence of the spine after steroid treatment **(H)**. **(I–L)** Histology of the stereotactic biopsy. Demyelinated areas are shown in Klüver-Barrera staining **(I)** and CNP-ase staining **(J)**, with numerous foamy macrophages [CD68, **(K)**]. Scattered CD45+ lymphocytes are present in the demyelinated areas **(L)**.

We present the case of a 47-year-old man with malignant melanoma who presented to the department of dermatology with a 6.5mm ulcerated melanoma of the occipital scalp. Molecular analysis revealed a BRAF V6000E mutation, NRAS wild-type and KIT wild-type. As the sentinel lymph node biopsy showed micrometastasis in the retroauricular region, the patient underwent left-sided neck dissection (level 2-4) with no sign of tumor spread. Staging, including MRI of the brain and combined whole-body positron emission tomography (PET) and computed tomography (CT) showed no indication of additional metastasis. According to the American Joint Committee on Cancer (AJCC) the staging at diagnosis was IIc and changed to IIIc after sentinel lymph node biopsy. Medical history was absent for autoimmune diseases, diseases for immunosuppression (e.g. HIV) or treatment with immunosuppressants. Also, the patient´s lab results showed no sign of immunosuppression at the time of diagnosis. The patient was offered treatment within CheckMate-915, a randomized, double-blinded adjuvant trial investigating nivolumab combined with ipilimumab compared to nivolumab by itself (https://clinicaltrials.gov/ct2/show/NCT03068455?term=checkmate+915&cond=melanoma&draw=2&rank=1). He started treatment on the combinatorial treatment arm with ipilimumab (1mg/kg, every 6 weeks) and nivolumab (240mg, every 2 weeks). Five weeks after treatment start the patient developed signs of cerebellar disease with dysmetria, pathologic heel-to-shin exam and gait ataxia. As his symptoms worsened over time, treatment was stopped after 2 cycles of ipilimumab and 3 cycles of nivolumab. A brain MRI conducted at that time showed multiple spot-shaped periventricular, leptomeningeal and parenchymal contrast enhancements suggestive of parenchymal and predominantly leptomeningeal metastasis (**Figures 1A–C**). MRI of the spine was consistent with leptomeningeal metastasis with nodular enhancement tracking along the cervical and thoracic segments and conus terminalis (**Figure 1D**). Subsequent cerebrospinal fluid (CSF) evaluation performed twice every other day showed a mildly elevated protein level and a moderate lymphomononuclear pleocytosis of 167/µl but absence of tumor cells on neuropathologic evaluation (**Table 1**, left column). Immunocytology revealed stimulated lymphocytes and activated monocytes; staining was negative for Melan A and HMB 45; no melanoma cells were detected. Additionally, no evidence of lymphoma could be found in a clonality analysis for B and T-cell receptors.

**Table 1:** CSF characteristics at time of diagnosis and follow-up.

CSF characteristic	Sample 1 (at diagnosis)	Sample 2 (after corticosteriod treatment)
Appearance	Clear	Clear
White blood cells/nl	132	31
Differentials	Mononuclear Cells, Lymphocytes	Mononuclear Cells, Lymphocytes
Protein (mg/dl)	122	124
Lactate (mmol/l)	3, 1	2, 4
Glucose (mg/dl)	58	68

Since the MRI was strongly indicative of leptomeningeal metastasis whereas CSF was negative for tumor cells, the patient was subjected to stereotactic biopsy of a right-sided contrast-enhancing periventricular lesion (**Figure 1C**). The neuropathologic evaluation showed white matter tissue with reactive gliosis and sharply demarcated demyelinated areas (Klüver-Barrera staining (**Figure1I**), positive staining for myelin protein CNPase (**Figure 1J**) and MBP, as well as dense macrophagocytic infiltrates (**Figure 1K**) and perivascular accentuated lymphocytic CD45^+^ (**Figure 1L**) infiltrates containing CD3^+^ and CD20^+^ cells of low to moderate density. The tissue was negative for lympho-blastoid B- or T-cells, melanosomal antigens (Melan A and HMB45) and clonality analysis. Additionally, there was no IDH-1 R132H mutation-specific stainability, nor alpha thalassemia/mental retardation syndrome X-linked (ATRX) loss or p53 accumulation. We designated this finding with the term pseudomeningeosis.

Assuming the clinical deterioration and findings evident on MRI were induced by ICI treatment, high-dose corticosteroid treatment was initiated (1g/d for five days) with consequent steroid tapering and prompt clinical improvement was observed. MRI and CSF re-evaluation were conducted six weeks later showing a near-complete remission (**Figures 1E–H**, **Table 1**, right column). Since discontinuation of ICI treatment, no other tumor directed treatment was initiated. On the latest follow up on 05/2020 there was no indication for tumor progression. The patient remains progression free for 22 months.

## Discussion

We here present the first patient with malignant melanoma, in whom biopsy confirmed checkpoint inhibitor induced demyelinating disease that radiographically could not be separated from periventricular, leptomeningeal and parenchymal metastatic disease. Subsequent corticosteroid treatment resulted in near-complete radiographic and clinical remission. As ICIs unmask self-tolerance and thus autoimmunity, immune-related adverse events have been reported. Even several cases of patients with similar findings under ICI therapy have been published in the literature (6, 7). However, histological confirmation was not obtained in any of these cases leaving uncertainty regarding the underlying diagnosis. Interestingly, ICIs have been described to induce demyelinating disease progression in patients with preexisting multiple sclerosis or radiographic isolated syndrome (8, 9). However, our patient had no white matter lesions prior to the start of treatment, leaving a possibility that ICI treatment triggered the emergence of white matter lesions.

Our case stresses the importance of a close interdisciplinary collaboration and rigorous diagnostic workup of patients treated with ICIs developing neurological symptoms and/or MRI findings. Only a biopsy led to the correct diagnosis of pseudomeningeosis and ruled out tumor metastasis. It remains to be elucidated whether non-invasive advanced imaging techniques such as amino-acid positron emission tomography may facilitate a diagnosis without the need for biopsy, as has been established with primary brain tumors in the setting of pseudoprogression (10). Pseudoprogression refers to treatment related changes on MR-imaging mimicking tumor progression with a subsequent decrease on tumor burden on follow-up imaging. The phenomenon is due to an increased immunostimulation. irNAEs have been described to occur early into treatment (5), as was the case in our patient who developed symptoms 35 days after ICI treatment start. As irNAEs have been reported with an incidence of 4% - 12% (4, 5) and it is likely that ICI treatment will surge in near future, treating physicians should become aware of this severe adverse event that responds well to steroid treatment to avoid premature discontinuation of an otherwise efficacious therapy.

## Conclusion

ICIs comprise a promising treatment option that is being used across many tumor types. Our case report stresses the importance of being wary of irNAEs that may mimic leptomeningeal dissemination. This is particularly crucial as irNAEs can be effectively treated with corticosteroid therapy.

## Data Availability Statement

The original contributions presented in the study are included in the article/**Supplementary Material**. Further inquiries can be directed to the corresponding author.

## Author Contributions 

Conceptualization: TS, SK, and MG. Data curation: TS, SK, EL, AJ, SZ, LL, CO, EC, DP, LZ, and MG. Investigation: TS, SK, EL, AJ, SZ, EC, DP, LZ, and MG. Validation: SK, EL, AJ, SZ, DP, KK, US, MS, CK, BS, LZ, and MG. Visualization: TS, SK, AJ, and SZ. Writing - original draft preparation: TS, SK, EL, and LZ. Writing - review and editing: all authors. All authors contributed to the article and approved the submitted version.

## Conflict of Interest

The authors declare that the research was conducted in the absence of any commercial or financial relationships that could be construed as a potential conflict of interest.

## Supplementary Material

Supplementary Figure 1 Timeline.
