# Supplementary figures and images for: Case Report: Pseudomeningeosis and Demyelinating Metastasis-Like Lesions From Checkpoint Inhibitor Therapy in Malignant Melanoma
Source: Front Oncol. 2021 Apr 15;11:637185. doi: 10.3389/fonc.2021.637185 (PMC8081911; doi:10.3389/fonc.2021.637185)

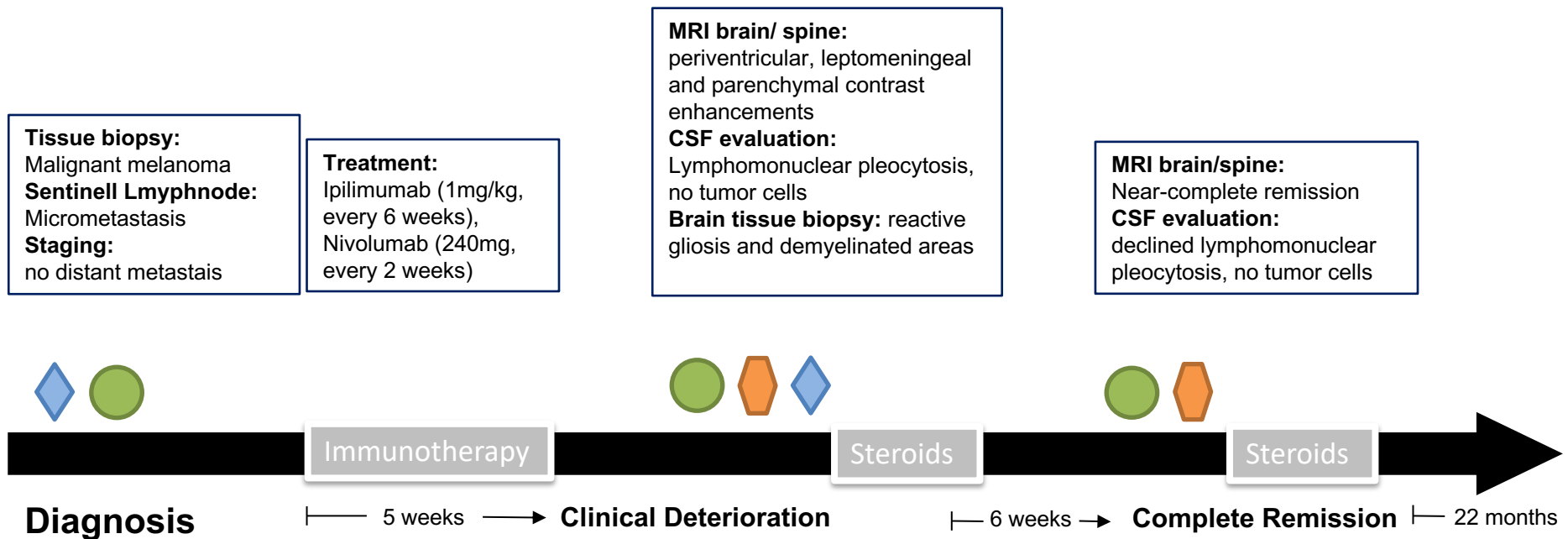

Supplement: Supplementary Figure 1 — Timeline. [file Image_1.pdf]
